# An Efficient Direction Field-Based Method for the Detection of Fasteners on High-Speed Railways

**DOI:** 10.3390/s110807364

**Published:** 2011-07-25

**Authors:** Jinfeng Yang, Wei Tao, Manhua Liu, Yongjie Zhang, Haibo Zhang, Hui Zhao

**Affiliations:** School of Electronic, Information and Electrical Engineering, Shanghai Jiao Tong University, Shanghai 200240, China; E-Mails: chengboyang@gmail.com (J.Y.); taowei@sjtu.edu.cn (W.T.); Manhua@sjtu.edu.cn (M.L.); zhangyongjie@sjtu.edu.cn (Y.Z.); haibozhang@sjtu.edu.cn (H.Z.)

**Keywords:** machine vision, fastener recognition, Direction Field (DF), LDA, pattern matching, VOSSLOH fastener

## Abstract

Railway inspection is an important task in railway maintenance to ensure safety. The fastener is a major part of the railway which fastens the tracks to the ground. The current article presents an efficient method to detect fasteners on the basis of image processing and pattern recognition techniques, which can be used to detect the absence of fasteners on the corresponding track in high-speed(up to 400 km/h). The Direction Field is extracted as the feature descriptor for recognition. In addition, the appropriate weight coefficient matrix is presented for robust and rapid matching in a complex environment. Experimental results are presented to show that the proposed method is computation efficient and robust for the detection of fasteners in a complex environment. Through the practical device fixed on the track inspection train, enough fastener samples are obtained, and the feasibility of the method is verified at 400 km/h.

## 1. Introduction

Nowadays, large railway networks need strong security mechanisms and must be maintained continuously to ensure safety. However, for both heavy work and objective requirements, trained personnel for railway maintenance cannot meet the needs of periodic inspections. Therefore, several automatic rail detection devices [1–3] are used, such as Japan’s East-I, Austria’s EM-250, Germany’s RAILAB, Italy’s Roger2000, France’s MGV, the American Ensco, and ImageMap’s track inspection car, which are all known for railway maintenance.

**Figure 1.**
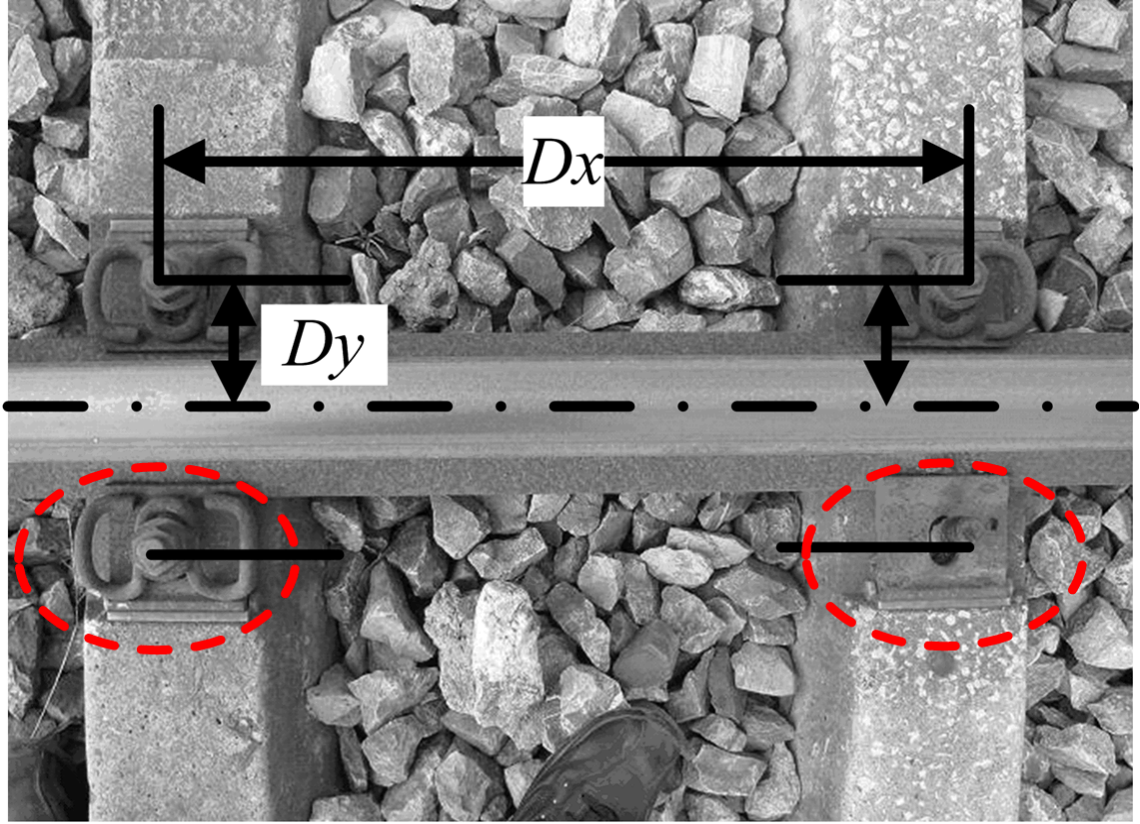
Sample image of fasteners.

A number of tasks are involved in railway maintenance, such as gauge measurement [4], track profile and wear measurement [5,6], ballast measurement [7], rail defect diagnosis [8], and concrete rail seat abrasion, among others. The current article focuses on fastener detection as an important part of rail inspection systems [5], which secures the rail to the sleepers or concrete base. Fastener detection is an important service involved in rail inspection. Our aim is to detect whether fasteners are present at the relevant location in a high-speed railway. In practical situations, the main kind of fastener used is the German VOSSLOH, as shown in Figure 1 (dashed line).

The current literature provides a few image-based methods for fastener detection. Hsieh *et al.* [9] applied a morphological approach to obtain the outline of fasteners, which can inspect them on the concrete or ballasted track to determine if they are broken and which has a 77% recognition rate for broken clips. It entails much computation time because of its time-consuming image processing tasks such as histogram analysis, open and close operation, *etc*. However, it cannot suppress large brightness variances and environment interferences and hence cannot meet real-time requirements. Stella *et al.* [10] and Marino *et al.* [11] used wavelet analysis and multilayer perceptron neural classifiers to detect hexagonal-headed bolts (a kind of fastener), revealing the presence/absence of fastener bolts with high detection and classification rates. However, the result is not applicable to our situation due to the complex shape and environment of the VOSSLOH fastener.

For VOSSLOH fastener specific detection, the current study aims to complete a fastener detection system with complex shapes in a complex environment. It introduces a rapid fastener detection method that completes high-speed noncontact detection based on the machine vision technique.

The environment of the fastener is complex, so its images are unstable. Sometimes, the edge of elastic clips for the fastener is obscured (as shown in Figure 1) because of surface erosion, dust coverage, motion blurring, and brightness fluctuations. Further, stones and other debris that cause occlusions are found. Therefore, simple brightness and edge features are not appropriate for recognition. Regional features are difficult to obtain. After extensive testing, we found that a certain scale texture of the fastener is stable, which is independent of brightness and robust for partial occlusion. Hence, we introduce a Direction Field (DF)-based method, which is commonly used to describe texture. The current paper uses a DF template of the fasteners for recognition, introducing Linear Discriminant Analysis (LDA) to obtain the weight coefficient matrix for matching. To limit the need for computation while guaranteeing stability, we use block DF with discrete sampling points.

The present work is organized as follows. Section 2 presents the main system overview. Section 3 focuses on some DF theories relevant for our application. Section 4 discusses the matching method, and Section 5 presents a number of tests to verify the feasibility of the DF descriptor and the matching stability. Section 6 introduces the disturbance experiment and the practical railway image results, and finally, Section 7 presents the conclusions and future perspectives.

## 2. System Overview

**Figure 2.**
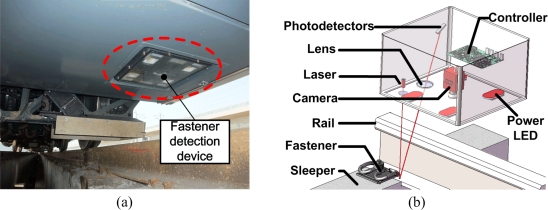
Detection system (**a**) and its composition (**b**).

**Figure 3.**
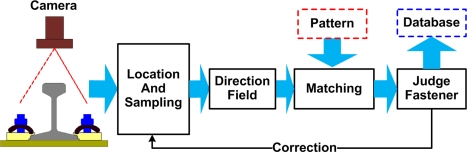
Schematic diagram of the fastener detection system.

The fastener detection system is shown in Figure 2, and the schematic diagram is shown in Figure 3. Two parts are distinguished:

First, a system locates where the fastener should be, drawing attention to the area of interest on the railway. It is a high-speed laser triangulation system [12] which consists of laser, photodiodes, and lens, as shown in Figure 2(b). Its main role is to obtain the approximate location of the fastener based on the height measurement of the sleeper or other fixed structure for the fastener. The optical and electronic system can transform the height signal of the fastener’s fixed structure to a trigger signal of the camera in order to obtain proper image samples.

Second, the fastener detection system, including a high-speed camera (AVT-GE680) and Light Emitting Diode (LED) strobe, aims to capture the images of the candidate area of the fastener, transfer these images to the processing unit, and then use the recognition algorithm to determine the status of fasteners. The camera receives trigger signals from the laser displacement sensor above. The short exposure and the high power LED lighting ensure that the image is clear and in good condition for image processing.

## 3. Direction Field

To detect fasteners along the railway at high speed, we install the detection devices on the track inspection vehicle. The detection speed may be up to 400 km/h. The fasteners we want to detect are shown in Figure 1. The fastener shape, posture, and orthogonal relative position to the rail (*Dy*) are stable, but the environment is complex and prone to corrosion. The quality of the image is unstable and blurred to a certain extent. For this high-speed detection, the low resolution, blurred image, and uncertainty of the environment are the difficulties that must be considered.

**Figure 4.**
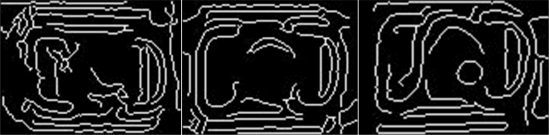
Canny edge detection for fastener images.

Some traditional detection methods, such as edge detection, are unsuitable for fasteners. Figure 4 shows some edge detection results from the original fastening image using a canny edge detector, in which the edge features are unstable and difficult to identify. Brightness feature and region feature [13] are also unsuitable and cannot be obtained easily. Rich local descriptors like shape context [14] and segmentation averaging [15] may provide robust matching for this kind of image, but they require too much time. Hence, we need other stable features; one good choice is texture because it is not sensitive to brightness, it is robust for some local disturbances, and it can obtain concise features at a certain scale.

For the current paper, considering the stability of the feature, we use DF for the feature recognition, which is an ideal feature for texture in complex environment [16], like fasteners. The reasons are as follows:

1. It is insensitive to light intensity and material surface state, such as brightness disturbance and surface corrosion which is universal in railway site. Reference [16] shows that DF is relevant to the proportion of horizontal gradient and vertical gradient, not the absolute brightness of the image.
2. It can suppress some noise higher than a certain frequency because of block optimal estimation [16].
3. The DF feature descriptor can avoid the instability resulting from occlusion, because it is a rich descriptor for the fastener [17]. Many descriptors weaken local disturbances.

Several methods for the extraction of DF exist. On the basis of the algorithm of Reference [16], we use the block DF based on discrete sampling.

The formula of DF is:

(1) $$\theta ={\text{tan}}^{-1}(\frac{\sum_{m=1}^{m=N_{1}}\sum_{n=1}^{n=N_{2}}\left(2\cdot {\mathrm{dx}}_{\mathrm{mn}}{\mathrm{dy}}_{\mathrm{mn}}\right)}{\sum_{m=1}^{m=N_{1}}\sum_{n=1}^{n=N_{2}}\left({\mathrm{dx}}_{\mathrm{mn}}^{2}-{\mathrm{dy}}_{\mathrm{mn}}^{2}\right)})/2+\pi /2$$

For image processing, *θ* is the optimal direction estimation of the relevant block; *m* and *n* are the relative coordinates of the image point in the block which is processed; *N_1_*, an *N_2_* are the width and the height of the blocks of which we need to obtain the directions; and *dx_mn_* and *dy_mn_* are the horizontal and vertical gradients of the relevant pixel point in the image. Generally, d*x* and d*y* can be calculated by the Sobel gradient, and the processing of DF is as follows:

1. Calculate the gradient of the image using the gradient operator.
2. Choose the sampling points and block size according to the texture.
3. Calculate the DF feature.

As discussed in reference [17], we also need a rich descriptor for robust recognition of the fastener. Taking DF as a global rich descriptor is robust and effective. It will be shown in the discussion that follows.

## 4. Fastener Detection Method

### 4.1. Obtaining the Feature Descriptor of the DF Method Using the Proper Parameters

**Figure 5.**
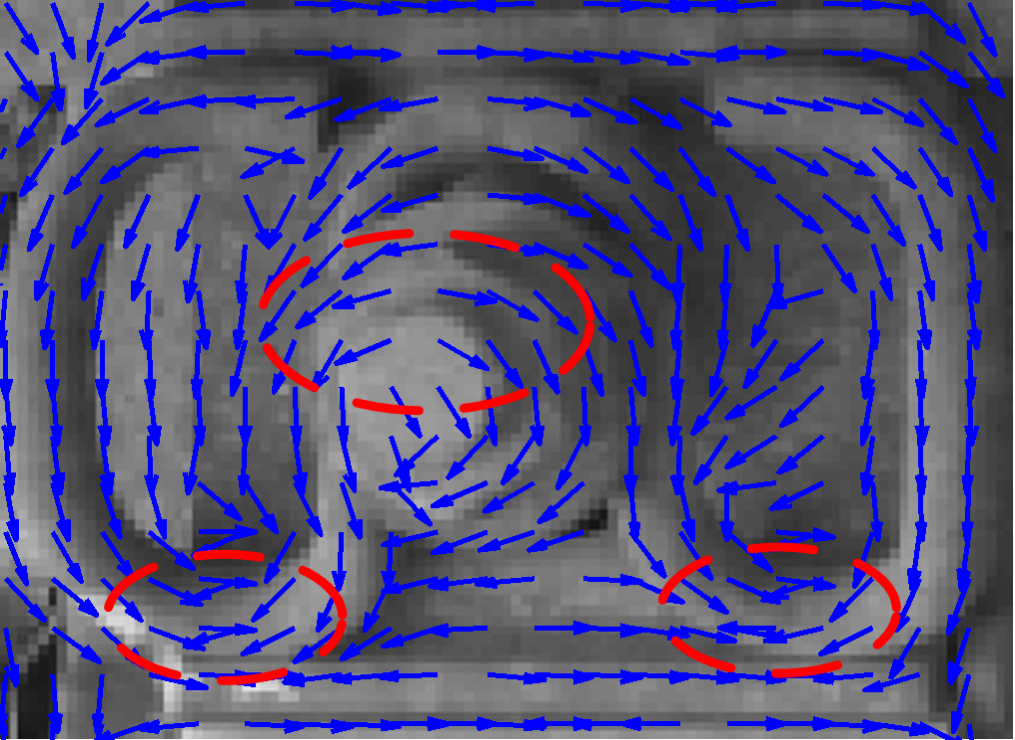
Direction Field demonstration.

As far as we know, the use of the DF method in fastener recognition is novel and inspired by fingerprint recognition [18]. The block DF can suppress the effect of brightness variation and high-frequency noise, which is common in the railway environment. In recognition processing, the use of an appropriate feature and feature template is important. Considering the region of the fastener and using the DF method, we can obtain a template composed by the DF element (Figure 5).

Then we can match the relevant DF features, completing the recognition. In application, we should determine two parameters, the size of the block and the interval of the DF sampling in the image, for the feature descriptor of the template and for sampling the image sequence as well.

**Figure 6.**
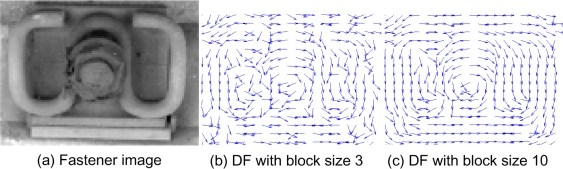
Image of the fastener and its DF. (**a**) Original fastener image; (**b**) DF with a block size of 3 × 3 pixels; and (**c**) DF with a block size of 10 × 10 pixels.

**Figure 7.**
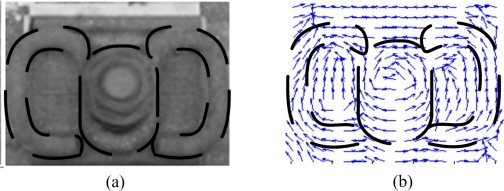
Image of the fastener (**a**) and its DF (**b**).

The block DF uses block smoothing and optimal estimation to reduce the effect of high-frequency noise and brightness variance. According to Rao [16], a “block” is a smoothing filter with a fixed width and height, as *N*_1_ × *N*_2_ of Equation (1). Different block sizes affect matching. If the size is large, many feature details are missed because of excessive smoothing. As a result, the matching precision becomes lower, and more mismatches may appear. On the contrary, a small block size cannot smoothen local noise like stones, dirt, and so on, which results in instability for feature extraction. Figure 6(b,c) shows the effects of different block sizes. According to Rao [16], the size of the block is associated with texture distribution. The diameter of the fastener clip is approximately 8 pixels in our image, and the main texture profile is the body of the fastener clip (Figure 7). Therefore, the block size should be bigger than 8 × 8 for smoothing.

To reduce the feature’s complexity, we use discrete DF elements for the feature descriptor on the basis of discrete sampling. Another important factor for the DF feature is sampling interval, which determines the feature details and affects matching stability. Experiments show that if the interval is 5 × 5 pixels with a block size of 10 × 10 pixels, then we can obtain suitable features and need little computation for matching.

In image processing, we use a big block to reduce the noise, apply suitable intensive sampling to ensure the stability of the feature descriptors. For this application, we use 10 ×10 pixel blocks around sampling points with 5 × 5 pixel intervals to obtain the DF features; that is, 10 × 10 pixels contribute to every relevant direction element, and the sampling is 5 × 5 pixel intervals. As shown in Figure 5, every DF element is an estimate direction of 10 × 10 pixels around the sample point.

### 4.2. Obtaining the DF Template of the Fastener

We use practical images with fasteners, focus on the region of the fastener, and extract manually a series of templates with a suitable window, as shown in Figure 6(a). Then we calculate the DF for every template to obtain the fastener feature descriptor. In practice, if the fastener DF of an individual is slightly different, we can use each as the pattern. However, the actual image is selected randomly, and each of the sample features has random noise that introduces an unstable factor. In addition, the DF of the pattern should represent the ideal pattern for the fastener and should smoothen the noise. In this regard, the computation of the arithmetic mean of all fastener DF templates is an effective method.

According to Equation (1), the value of DF is (−π/2 ∼ π/2). We should take into account that the opposite direction can offset the result. As shown in Figure 5 (dashed ellipse), the approximately opposite direction represents a similar texture direction for the image. To maintain the consistency of the direction, we use the method of opposite correction for every sample and complete the average of DF templates in the iteration.

**Figure 8.**
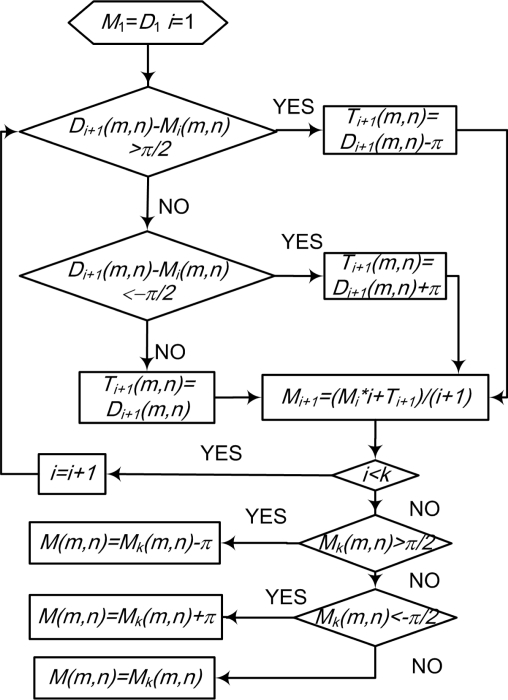
Flowchart for the average of the DF template.

We assume that the initial DF template sample set is *D*, which is a source to form the final template of the fastener. They are a series of DF matrix trix elements as shown in Figure 5 (*D*_1_ ∼ *D_k_* ), we assume that the number of samples is *k*, and the resulting fastener template we want is *M*, which is an ideal feature descriptor for the fastener. We then use the first DF matrix of *D* as the seed (that is to say, *M*_1_ = *D*_1_) and *T* as the corrected matrix for every sample processing. Using the iterative average method in Figure 8, we can get an ideal DF template of fastener after opposite direction correction and average (/(*i* + 1)). *i* is the number of the sample, (*m, n*), mean the corresponding direction field elements coordinates in template. For ease of expression, we take (*m, n*) as a representative, and in fact they traverse all the angle elements of feature.

After computing for the arithmetic mean with opposite correction, we can obtain an ideal fastener pattern, which is an ideal DF matrix in mathematical expression. Then we can calculate the matching distance for recognition. Without loss of generality, we can use the absolute distance. However, for practical matching, we should highlight the part of the fastener clip and suppress the disturbance of other areas (Figure 7, dashed line show the interesting part of fastener image), so that the weighted absolute distance is better.

### 4.3. Computing the Weight Coefficients

Images that contain the fastener can also be divided into two parts: the region of the fastener body and other parts (Figure 7). We should weaken the effect of certain disturbances in the region that are not the fastener body, as well as strengthen the fastener parts to obtain high precision and robustness. This is because the main part we focus on is the fastener. Intuitively, we should focus on the weight factor in the region of interest, which is the fastener, using two methods. One is artificial assignment under the mask template, which is similar to “Chamfer Matching” [19]: different regions have different weights assigned, and a refinement of the weight template is obtained. Figure 8(a,b) shows the fastener image and it’s DF; the dashed line refers to the area of the fastener clip which we should focus on. The other method is referred to as the sophisticated classification method, such as the neural network or statistical method in obtaining the proper weights for matching. We can take this weight matrix as a coefficient vector, then use LDA [20–22] to produce this coefficient vector (CV) based on two classes of DF template (fastener and missing). CV best discriminates between the two classes and is the weight coefficient matrix that maps the DF matrix to the status space of the fastener. It performs excellently for fastener matching. We use LDA to obtain a reasonable weight coefficient matrix. It can highlight the distinction between the two cases of with fastener and without a fastener, completing the mapping from the DF of the image to the status space of the fastener. The detailed calculation process in shown in reference [20].

### 4.4. Matching of the Fastener

We discuss the matching process in this section. We obtain the pattern, the weight coefficient matrix, and the image samples to be identified, measuring the orientation coherence according to the method of Rao [16]. If much coherence exists, the fastener is present and *vice versa.*

To estimate coherence, we can use the comparison of the direction angle Equation (2) or a comparison of the trigonometric function value of angle Equation (3) similar to the method of Rao [16]:

(2) $$D_{i}=\sum_{j=1}^{n}\sum_{k=1}^{m}w(j,k)\cdot \text{min}(\left|M(j,k)-S(j,k)\right|,\pi -\left|M(j,k)-S(j,k)\right|)$$

(3) $$D_{i}=\sum_{j=1}^{n}\sum_{k=1}^{m}w(j,k)\cdot \left|\text{cos}(M(j,k)-S(j,k))\right|$$

where *n* and *m* are the row size and the column size of the DF matrix, respectively, and (*j,k)* is the direction field angle element coordinates, *M* is the pattern matrix, *S* is the sample’s DF matrix that is to be identified as derived from the detection image sequence, *w*(*j,k*) is the weight coefficient for a certain fastener DF template element, and *D_i_* is the distance between the pattern and sample *i*, which is used to estimate the status of the fastener.

## 5. Feasibility Verification

The test environment is considered ideal in terms of avoidance of disturbance because the condition of the real test railway is built for 400 km/h. We cannot obtain diverse samples from this. Hence, for the general test, we use a Canon SX110 IS digital camera (based on 1/2.3″ CCD, 3,456 × 2,592 pixels, F/3.2) and collect images at the railway site under natural conditions. We can obtain more samples in different conditions with the help of railway maintenance workers, which have better diversity than actual samples. Figure 1 shows the image of the fasteners, which we use to verify the feasibility of our method. For processing, we resize the image to 640 × 480 on the basis of the high-speed camera’s resolution of AVT-GE680.

### 5.1. Ability to Divide the Candidate Image Region into the with Fastener and without a Fastener

**Figure 9.**
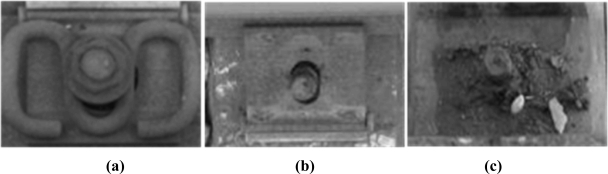
(**a**) Fastener image; (**b** and **c**) Fastener missed.

We collected 300 fastener images and 100 images without a fastener with the help of railway maintenance workers. We focus on the region of the fastener and extract manually a series of candidate areas by a proper window. We divide these into two classes, with a fastener and without a fastener, as shown in Figure 9.

Their DF descriptors are obtained, and the absolute angle difference value is used to calculate intra and interclass distances. For comparison, we also calculate intra and interclass distances with the absolute trigonometric function difference value. Here *w*(*j*, *k*) = 1, just verify the feasibility of DF feature descriptor. Table 1 shows the results of the maximum and minimum of the intra and interclass distances using the absolute distance with Equation (2). Table 2 shows the results using Equation (3).

The results show that the intraclass distance is smaller than the interclass distance for both images with and without a fastener. Therefore, the classes are divisible, and the recognition of fasteners is feasible. No evident differences between the two methods (Equations (2) and (3)) are found, and for easy application, we use the absolute angle difference value (Equation (2)) to calculate the distance directly.

Subsequently, coefficients are calculated between the fastener class and the without a fastener class, and coefficients *w*(*j*, *k*) are added to the distance calculation. Table 3 shows the results of the maximum and minimum of the intra and interclass distances by weighted distance on the basis of absolute distance.

In Table 3, the distance is corrected by the appropriate proportionality because LDA introduces a large normalization factor. The maximum distance of interclass is equal to Table 2. Table 3 shows that the intra and interclass distances ratios are smaller than before because of the focus on the key area. The ratio of the fastener intraclass maximum distance to the interclass minimum one is 46.2/62.0 = 0.745 (0.779 in Table 1). Some disturbance may appear because the sample number is limited. To some extent, the weighted distance can suppress disturbance.

**Figure 10.**
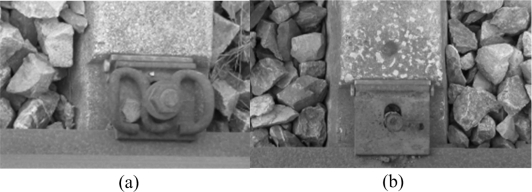
Image samples for fastener matching. (**a**) With a fastener; (**b**) Without a fastener.

**Figure 11.**
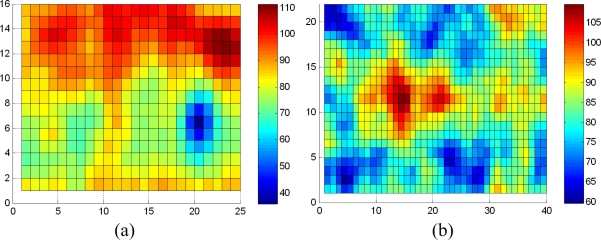
Matching and searching results from Figure 10 using the relevant DF pattern. (**a**) With a fastener; and (**b**) Without fastener.

For application, we use the average of fastener sample intraclass maximum distance and the interclass minimum one as a threshold to determine the status of fastener. If the matching distance is little than that, fastener is presence, and vice versa. In practical, complex environment bring more disturbances. For example, as a matching result of Figure 10(b), in Figure 11(b), some matching distances are lower than 62. Those can be excluded based on location information. Nevertheless we realize that some disturbances are unpredictable, because our limited samples can’t cover all cases. There must be some matching distance that fell into range of 46–62, so we must adopt a reasonable threshold through more samples and pertinent requirement for certain detection. We could combine other information, such as location and weather.

### 5.2. Matching Fasteners in Image

To a certain extent, in the sample image (Figure 10), the fastener location is not accurate because of high speed, and we cannot extract the exact location candidate that contains the fastener. Therefore, we should search for the fastener in the approximate position of the image on the basis of the positioning signal that we can obtain by the laser triangulation system and velocity signal. We then determine the presence or the absence of the fastener. The fastener DF pattern is taken as the matching factor, and the whole image or a location window is matched on the basis of the absolute distance (e.g., images with or without fasteners; Figure 10).

We use the fastener pattern and the corresponding coefficient matrix to match the fastener in the whole image as shown in Figure 10 (for the results, refer to Figure 11). The color bar shows the distance scale, and horizontal and vertical coordinates indicate the location of matching. We can find a matching area in Figure 11(a) obviously. There are some matching distances below 62 for Figure 11(b), refered in Table 3, that can be excluded by thresholding or location information. Results show that the DF feature descriptor is suitable for matching.

### 5.3. Influence of Motion Blur

**Figure 12.**
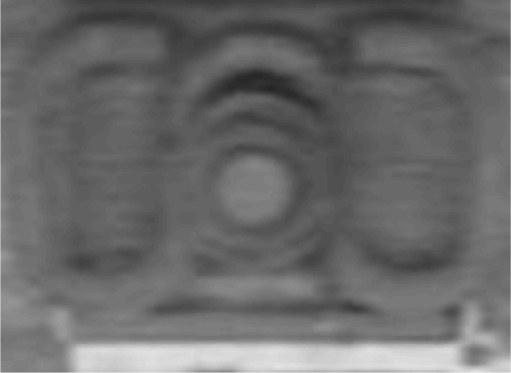
Simulation of an image with a motion blur.

Motion blur is inevitable because of the high speed. Figure 12 shows the motion blur along the railway mainly. In practical application, the shutter time is 25 μs, and the speed of the train is 400 km/h, so the motion blur effect distance is approximately 3 mm. This is roughly 3 pixels in our application. Motion blur weakens the edge, increasing the difficulty of recognition. For the images in Figure 9(a), according to practice, we use the motion filter *F* = [0.125, 0.25, 0.25, 0.25, 0.125] to add some motion blur, as shown in Figure 12.

**Figure 13.**
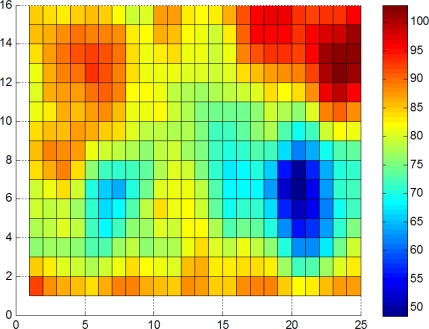
Matching result after adding motion blur.

Motion blur just weakens the edge. For the DF, the small blur effect is tolerable because of large-scale smoothing. To test this method for a blurred image, we add blur to the samples as shown in Figure 9 and compare the maximum and minimum of the intra and interclass distances with weight coefficients. The results are shown in Table 4. Meanwhile, we verify the matching capability using the same image (Figure 10(a) after adding motion blur (see Figure 13 for the results). Result shows that we can also find a matching area evidently, and matching is stable.

### 5.4. Comparative Experiment

We compare our method with Marino’s method (intuitively a better one) using several samples. We have 300 sample fasteners and 100 ones without a fastener (Figure 9); 50% of the samples in every class are set as the train set, and the other 50% is set as the test set. Therefore, there are 150 test samples with a fastener, and 50 samples without a fastener. The recognition rate is shown in Table 5. The results show that our method can achieve a higher recognition of 100% than Marino’s 72%, and is stable considering the complex shape and environment involved. Other than Marino’s method aimed at hexagonal fasteners, our proposed method is considered suitable for complex cases.

For Marino’s method, the main function of DWT (Discrete Wavelet Transform) is to reduce the dimension of the bolt image, which is effective for simple shapes and for good conditions. However, much noise and uneven brightness lead to incorrect recognition. As for Hsieh’s approach [9], it relies on edge and brightness. It cannot suppress larger brightness variances and environment interferences, so it is difficult to get appropriate features, as shown in Figure 5. On the other hand, morphological algorithms are used, which needs a lot of computation time. For one image of 320 × 240, the computation time is about 120 ms, while ours is 10ms. Because there are no stable features based on Hsieh’s approach, so the True-Positive ratio lower than 77% as they claim. One reason is that our test images are in complex environment.

## 6. Experimental Results for Disturbance and the Practical Image

### 6.1. Stability for Brightness Variance

**Figure 14.**
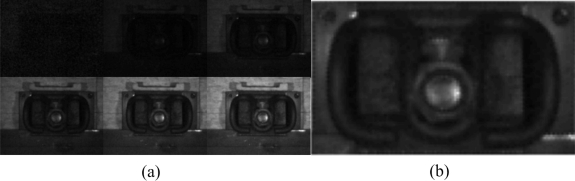
Fastener image in different light intensities and a normal pattern. **(a)** Fastener image sequence; **(b)** A fastener image as the pattern

The texture direction of the image is insensitive to brightness [16], so a DF that is stable in terms of light intensity and some brightness variance is acceptable. In practice, the image brightness should be within a feasible range, which is within a wide range for our method. In this section, we verify the stability of the matching under different lighting conditions. We obtain a fastener image sequence in the laboratory with increasing current for High power LED. The image sequence is shown in Figure 14.

**Figure 15.**
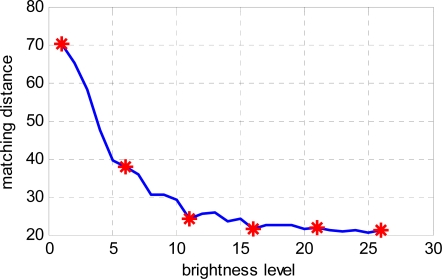
Matching distance for the image sequence in different light intensities.

We have a total of 26 images in different light conditions. Figure 15 shows the matching results using a normal DF pattern derived from that shown in Figure 15(b). The abscissa shows the brightness level of the image sequence, and the ordinate shows the minimum matching distance of the pattern and the sample image.

Results show that DF is not sensitive to brightness, except in extreme cases. In this curve, 26 points are found; “*” indicates the corresponding point for Figure 14.

### 6.2. Verification of the Capability for Noise Suppression and Tolerance

**Figure 16.**
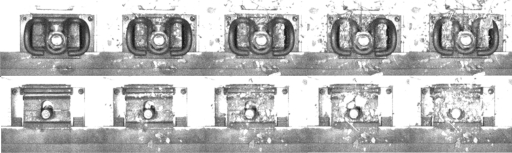
Fastener image with disturbance.

In the practical railway environment, some debris, stones, or leaves that affect the detection process can be found, so we should verify the stability of our method in terms of these disturbances. In the experiment, we add some soil and stones on the fastener, increasing the disturbance of the image and comparing the matching results in different conditions. The images of the experiment are shown in Figure 16. The upper row contains the images with fasteners, and the lower one contains those without fasteners. The matching distance is shown in Table 6, with Levels 1–5 corresponding to the pictures from left to right in Figure 16. They show an increase in the degree of disturbance.

We can see that disturbance has some effect on matching. Nevertheless, a small amount of disturbance is tolerable. Furthermore, evident differences for images with and without fasteners are found based on the threshold of 52 (referred in Table 3, (62 + 46)/2 = 52), which verifies that our method is feasible for practical fastener detection.

### 6.3. Practical Application Results

**Figure 17.**
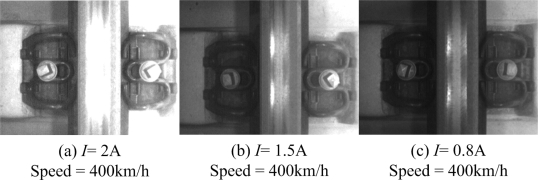
Practical images of fasteners at 400 km/h.

Recently, we have installed our device on track inspection trains for the China high-speed railway. Figure 2(a) shows our device, and Figure 17 shows the image samples for the fastener at 400 km/h and under different lighting conditions. *I* is the current of High power LED. We obtain fastener images covering about 100 km at an average velocity of 400 km/h. The matching results show that our device can match 99.99% fasteners appearing in the image and can meet speed requirements. The recognition rate is shown in Table 7. There are three conditions with different illumination (LED current). In Table 7, we get 100 km samples, so the fastener number of different condition is about 140,000.

We use a C++ programming environment, on a double core T6500@2.1 GHz CPU. With two scales at fasteners of 140 × 100 × 2 pixels, the image area is enough to serve as the matching base on our positioning system. The computation consumption of DF is 3.5 ms, the matching time is about 1 ms, the interval of the fasteners along the rail is about 650 mm, and the camera AVT-GE680 can complete 380 frame per second collections with 320 × 240 pixels. That is, if we use two processes of this case for the two rails, we can complete the online inspection at 510 km/h. In practical application, benefits from the use of GPU (Graphic Processing Unit) [23] and real-time detection can be obtained easily.

In this experimental phase, the railway is designed for high-speed railway of 380 km in the Xuzhou section of Beijing-Shanghai high-speed rail. It is new, so there are few disturbances. Results are good under good conditions. It shows that our method is suitable for fastener detection on this railway section. For 2A LED Current, the True-negative is caused by interference of sun. And for 0.8A LED Current, there is some disturbance due to insufficient light. So we found the good illumination for this railway should be about 1.5A LED Current.

## 7. Conclusion

We have presented an efficient image-based method for the detection of fasteners on high-speed railways. The DF is extracted as the feature descriptor, which is more stable in different conditions and can suppress some disturbances. The orientation coherence is used for matching the fasteners. The experimental results from the fastener images of the real railway environment prove the effectiveness and time efficiency of the proposed method. In future studies, we will enhance our method for classifying and recognizing different kinds of fasteners. We will generalize the application of our proposed method to other kinds of fasteners, as well as for other rapid detection applications in a complex environment.

## Figures and Tables

**Table 1.** Intra and interclass distances using the angle absolute difference value.

**Status**	**Minimum**	**Maximum**
Fastener–Fastener	0	**112.6**
Fastener–Missing	**144.5**	199.3

**Table 2.** Intra and interclass distances using the cosine function.

**Status**	**Minimum**	**Maximum**
Fastener–Fastener	0	**43.64**
Fastener–Missing	**56.8**	92.9

**Table 3.** Intra and interclass distances using Equation (2) with weight coefficients.

**Status**	**Minimum**	**Maximum**
Fastener–Fastener	0	**46.2**
Fastener–Missing	***62.0***	92.9

**Table 4.** Intra and interclass distances for the blur samples.

**Status**	**Minimum**	**Maximum**
Fastener–fastener	0	**60.2**
Fastener–Missing	68.1	92.9

**Table 5.** Recognition rate using our method and Marino’s method.

	**True-positive (TP)**	**False-positive (FP)**	**True-negative (TN)**	**False-negative (FN)**
Our method	150	0	0	50
Marino’s method	107	12	43	38

**Table 6.** Matching distance for Figure 14.

**Status**	**Level 1**	**Level 2**	**Level 3**	**Level 4**	**Level 5**
Fastener	21.94	25.81	24.25	32.08	39.31
Without fastener	63.06	60.63	57.05	59.01	65.37

**Table 7.** Recognition rate of practical images using our method.

**LED Current**	***I* = 2A**	***I* = 1.5**	***I* = 0.8A**
Total fastener number	14373	13657	14532
True-positive (TP)	14372	13657	14529
False-positive (FP)	0	0	0
True-negative (TN)	1	0	3
False-negative (FN)	0	0	0
Recognition rate	99.99%	100%	99.99%
